# Correlation of the CT Compatible Stereotaxic Craniotomy with MRI Scans of the Patients for Removing Cranial Lesions Located Eloquent Areas and Deep Sites of Brain

**DOI:** 10.3889/oamjms.2015.027

**Published:** 2015-02-12

**Authors:** Salih Gulsen

**Affiliations:** Baskent University Medical Faculty Hospital - Neurosurgery, Maresal Fevzi Cakmak cad. 10. sok. No: 45, Ankara 06540, Turkey

**Keywords:** Brain Tumour, eloquent areas, motor cortex, Computerized tomography, Stereotaxy

## Abstract

The first goal in neurosurgery is to protect neural function as long as it is possible. Moreover, while protecting the neural function, a neurosurgeon should extract the maximum amount of tumoral tissue from the tumour region of the brain. So neurosurgery and technological advancement go hand in hand to realize this goal.

Using of CT compatible stereotaxy for removing a cranial tumour is to be commended as a cornerstone of these technological advancements. Following CT compatible stereotaxic system applications in neurosurgery, different techniques have taken place in neurosurgical practice. These techniques are magnetic resonance imaging (MRI), MRI compatible stereotaxis, frameless stereotaxy, volumetric stereotaxy, functional MRI, diffusion tensor (DT) imaging techniques (tractography of the white matter), intraoperative MRI and neuronavigation systems.

However, to use all of this equipment having these technologies would be impossible because of economic reasons. However, when we correlated this technique with MRI scans of the patients with CT compatible stereotaxy scans, it is possible to provide gross total resection and protect and improve patients’ neural functions.

## Introduction

Intracranial tumours located at the eloquent areas and deep sites of the cerebrum have been difficult to treat surgically because of their high complication risks, including hemorrhagic infarct, cerebral tissue damage and bleeding [1-3]. Because of these damages, patients experience motor and sensory deficit, motor dysphasia, sensory dysphasia and various symptoms and signs related to damaged sites of their cerebrum [1-3]. So to prevent these complications, stereotactic resection of these tumours has been used for more than fifty years [4-8]. While performing stereotactic craniotomy, there are fundamental advantages. These are firstly, making intersulcal incison or if it is possible to remove any tumour without touching cortical part of the cerebrum. Secondly, making less retraction to the brain provides less damage to brain tissue [4-10]. To demonstrate the effectivity of the stereotactic craniotomy, we made nine consecutive stereotactic craniotomy in eight patients having intracranial mass located eloquent areas or deep parts of the brain with various pathologies, including glioblastoma, meningioma, abscess and cavernoma.

## Material and Methods

### Patient characteristics

Our series comprises nine craniotomies in eight patients with tumours at the cerebrum. Of these patients, brain tumours were settled in the eloquent cerebral regions -motor cortex, Heschl gyrus- or deep parts of the cerebrum. The mean age of these patients was 56 and a standard deviation of 9.41. The age range was between 41 and 63. For their intracranial mass lesions, these patients underwent craniotomy and tumour removal surgery. Moreover, we did these operations using computerized tomography assisted an arc-based CT compatible stereotactic frame system (Fischer ZD, Germany) at Baskent University medical center between 2013 and 2014. We obtained all information regarding preoperative and postoperative clinical, radiographic, histopathologic, and operative records from the online archive of the Baskent University Medical Faculty Hospital. Four of the patients were women, and four of them were men. The diagnoses were glioblastoma (n=2), metastatic non-small cell lung carcinoma (n=3), cavernous malformation (n=1), grade 2 meningioma (n=1), purulent abscess (Table 1).

**Table 1:** This table is showing the characteristics of the patients and theirs lesion location in the cerebrum. Besides their clinical condition in preoperative and postoperative period, it shows the result of their surgical operation.

Cases	Gender	Age	LL	LD (cm)	Pathologic Diagnosis	Preop. Exam.	Postop. Exam	Result
Case 1	F	60	RMC	5x6x5	Grade 2 Meningioma	LH	Improved**	GTR#
Case 2	F	63	LMC	3x2x2.5	Cavernoma	RH	Improved**	GTR#
Case 3	F	58	LMC	3x2x3	Glioblastoma	RH	Improved**	GTR#
Case 4	M	62	LPL (DL)	2x2.5x2.5	Glioblastoma	RH	Improved**	GTR#
Case 5*	F	60	RMC + LAPR	3x3x2.5- 3x4x3	Metastatic Lung Tumour	LH	Improved**	GTR#
Case 6	M	41	LPL (DL)	4.5x3.2x3	Metastatic Lung Tumour	LH + GD	Improved**	GTR#
Case 7	M	63	RTFR	3x2.8x2.8	Metastatic Lung Tumour	LH	Improved**	GTR#
Case 8	M	41	RMC	3x2x3	Pyogenic Abscess	LH	Improved**	DA#

Abbreviations: LL: Lesion localization; LD: Lesion dimension; Preop.Exam: Properative examination; Postop. Exam: Postoperative examination; F: Female; M: Male; RMC: Right motor cortex; LMC: Left motor cortex; LPP: Left posterior parietal; LAPR: Left anterior part of parietal lobe; DL: Deeply located; LPL: Left parietal lobe region; LTP: Left temporal lobe; RTFR: Right temporofrontal region; LH: Left hemiparesis; RH: Right hemiparesis; GD: Global dysphasia; GTR: Gross total resection of the tumour; DA: Drainage of the abscess.

* Case 5: This patient had seven metastatic lesions. Two of them were extracted by stereotactic method, and the third one located at the right side of the posterior fossa, and it was located superficially, so the third one was extracted by using a conventional neurosurgical procedure. The rest of the lesions-each of them- were smaller than 3x3x3 cm, so we decided to give radiotherapy to them.

** None of the patients experienced any new neurologic deficit. Furthermore, all of them showed better motor score and better neurological status.

# Preoperative and postoperative cranial MRI and/or cranial CT- from Figure 1 to Figure 12- showed no residual tumour or abscess at the operation site in all patients.

### Technique

We pick up preoperative cranial magnetic resonance imaging (MRI) and cranial computerized tomography (CT) with contrast from all patients. Following this step and finishing of the surgical procedures related to anesthesia, We selected patients having a mass located eloquent areas of the cerebrum or deep parts of it for stereotactic surgery for tumour removal. The we transferred the patients to our operation room. After endotracheal intubation and anesthesia, we settled stereotactic headholder, which is CT compatible equipment, to the patient’s head. We then transferred the patient to the CT unit to take contrast-enhanced CT with stereotaxy frame. Following making contrast-enhanced cranial CT with stereotaxy frame, we again transferred the patient to the operation room. Then, we moved the patient on the operation table. Then, we fixed headholder, that we previously settled on patient’s head, (Fischer ZD, Germany) to the operation table. Meantime, we calculated the target points from the taken cranial CT. Moreover, we compared this CT scans with MRI scans to decide the place of craniotomy and the cortical incision place to provide optimal conditions during the microsurgical tumor removing. Then we used this target point to determine craniotomy place, the cortical incision place and the deepness of the lesion. After craniotomy and determination of the cortical incision area and deepness of the lesion, we removed the lesion with microsurgical technique by Zeiss neurosurgical microscope (Oberkochen, Germany). Following the operation, we took cranial MRI and cranial CT from each patient. Moreover, we used cranial CT for evaluating any hemorrhage and cranial MRI to determine residual tumour at the operation site of the cerebrum.

## Results

We followed the vital signs including pulse, blood pressure, blood oxygen saturation, respiration rate and pattern, and neurological status after the operation. Also, we examined the patients after one hour, two hours eight hours, twenty-four hours of the operation. One day later we examined the patients two times in a day, and we discharged all patients within one week. None of the patients showed any deterioration regarding their vital signs. However, neurologic status of the patients was unstable in first two examinations. At the third examination, they showed stable neurologic examination compared to preoperative period. It was noteworthy that all patients showed neurological improvement twenty-four hours later of the operation. For example, all patients with hemiparesis could walk independently and to use their hand for daily activities. However, one patient with the diagnosis of abscess at the right motor cortex showed no improvement his skillful motor activity of the left hand. Though, his motor control of left shoulder and arm much better than of preoperative period. Another example is that the patient with left temporofrontal mass lesion- diagnosed as metastatic lung tumour- this patient’s global dysphasia was better than that of preoperative period. He was able to express and understand, but he had difficulty to find words in expressing his ideas. Except two of these patients, six patients showed almost complete recovery regarding their neurological status.

### Surgical outcome: preoperative and postoperative MRI and CT findings

**Figure 1:**
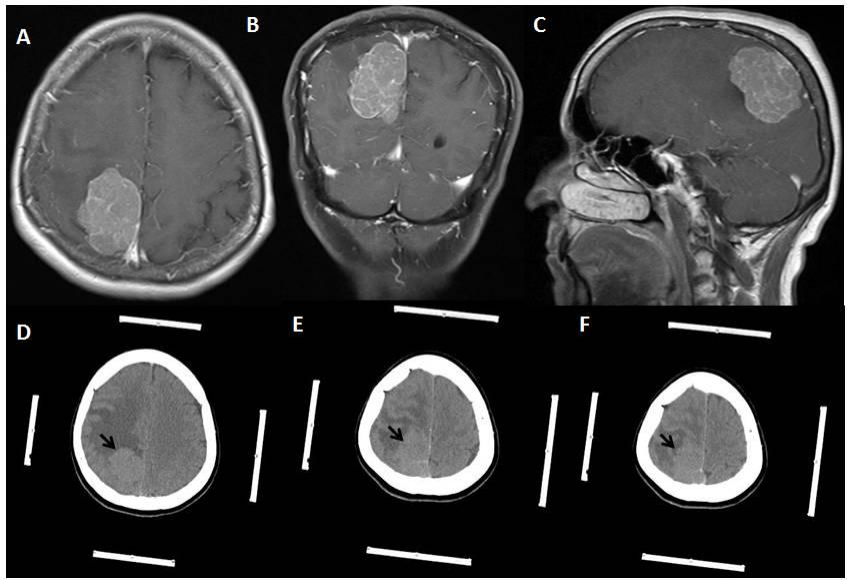
*A, B, C: (Table 1: Case1, Grade 2 meningioma): T1 weighted gadolinium-enhanced axial magnetic resonance imaging (MRI) scans showing right frontoparietal mass located just next to the superior sagittal sinus and extended from right parietal lobe to the right frontal lobe’s precentral gyrus (motor cortex). D, E, F: Contrast-enhanced cranial tomography showing right frontoparietal mass located just next to the superior sagittal sinus and extended from right parietal lobe to the right frontal lobe’s precentral gyrus (motor cortex). The white strips at the sides of the images are the marker of the frame based the stereotaxic system*.

We picked up all patients’ cranial CT and cranial MRI at the preoperative and the postoperative period. We took postoperative CT and MRI from the patients within three days of the operation. We took all patients’ contrast-enhanced MRI, and non-contrast cranial CT showed no residual tumor and hemorrhage causing pressure effect on the brain. Likewise, at the postoperative period of the T2 weighted cranial MRI, edema around the lesion was less than in the T2 weighted MRI (Fig. 1 – Fig. 8).

**Figure 2:**
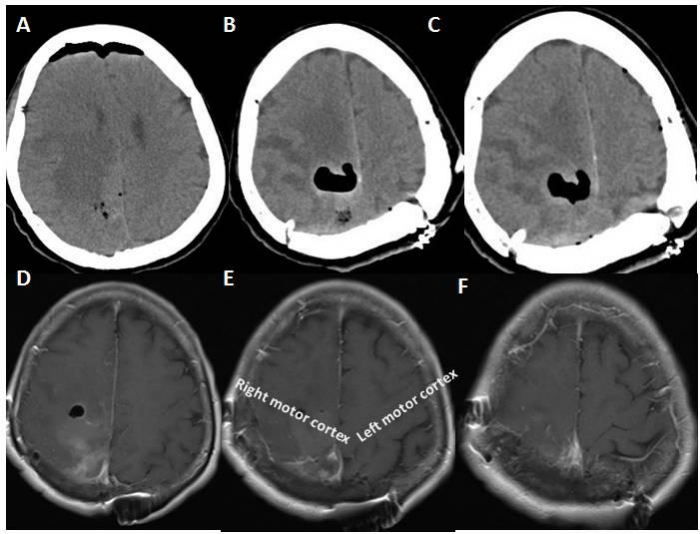
*A, B, C: (Table 1: Case 1, Grade 2 meningioma): Noncontrasted cranial computerized tomography was taken 24 hours after the operation. It shows no residual tumour and hemorrhage at the operation site being right parietal and posterior frontal lobe region. D, E, F: T1 weighted gadolinium-enhanced axial magnetic resonance imaging (MRI) scans showing right frontoparietal operation region and hypointense oval- shaped area at the right motor cortex. And there is no residual tumour this region*.

**Figure 3:**
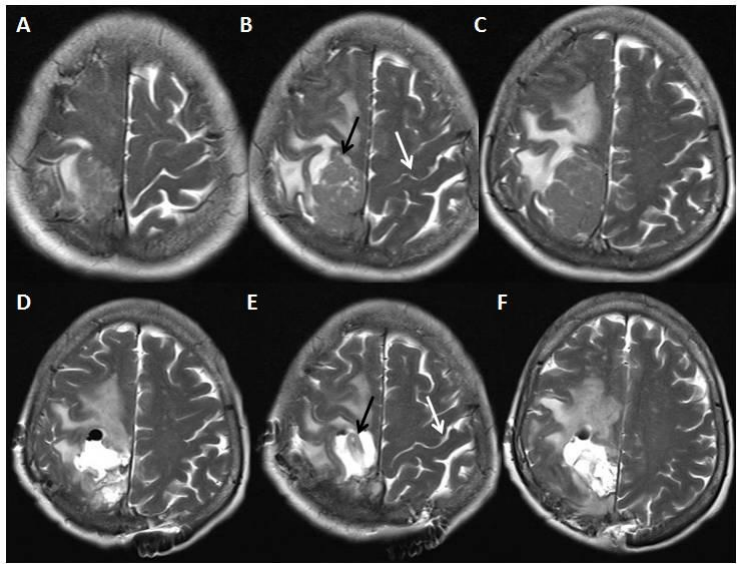
*A, B, C (Table 1: Case 1, Grade 2 meningioma): Noncontrasted T2 weighted cranial Magnetic resonance image –it was taken in the preoperative period- scans showing hypointense mass lesion occupied the right motor cortex. Moreover, black arrow showing invasion of the right precentral gyrus (right motor cortex) and anterior part of the right parietal lobe. White arrow is showing the intact left precentral gyrus (motor cortex). D, E, F: Noncontrasted T2 weighted cranial Magnetic resonance image –it was taken in the postoperative period- scans showing hyperintense area at the region of the right precentral gyrus and anterior part of the parietal lobe. Black arrow shows this area, and on the left hemisphere, white arrow indicating left precentral gyrus (motor cortex)*.

**Figure 4:**
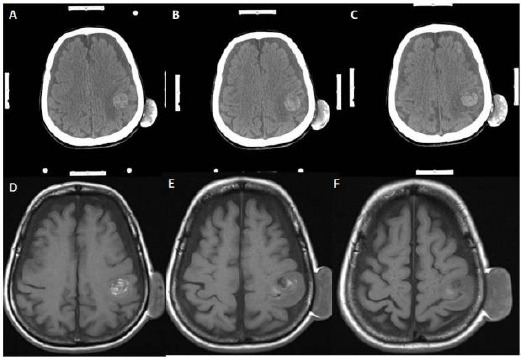
*A, B, C (Table 1: Case 2, Cavernoma): Noncontrasted computerized cranial tomography showing that hyperdense oval shaped lesion having hypodense areas within it. D, E; F: Noncontrasted T1 weighted axial cranial magnetic resonance image scans showing the heterogenous intensity and edematous appearance at the left motor cortex. Also, trichilemmal cyst is shown at the scalp these scans*.

**Figure 5:**
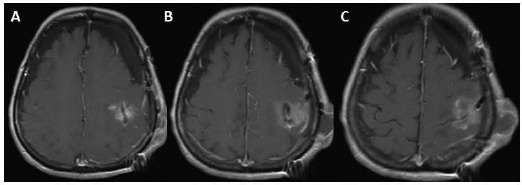
*A, B, C (Table 1: Case 2, Cavernoma): Gadolinium-enhanced T1 weighted axial magnetic resonance image scans taken at the postoperative period showing no residual lesion, but there is some postoperative hyperintense changing due to gliosis at the site of the operation. Also, it is noteworthy that we excised the trichilemmal cyst, so it is not seen on the scans*.

**Figure 6:**
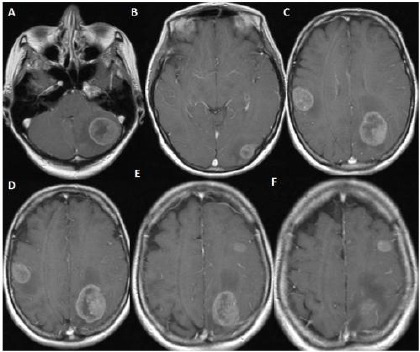
*A, B, C, D, E, F (Table 1: Case 5, Metastatic lung tumour): Gadolinium-enhanced T1 weighted axial magnetic resonance image scans showing seven different metastatic lesion with a heterogeneous intensity. I extracted three of seven lesions, because three of them larger than 3 cm on each scan and smaller lesions were left for gamma knife surgery. The first one of the extracted tumour located at the left side of the posterior fossa and it was extracted through conventional craniotomy technique, so it is out of interest of this article. The second one of them located at the right motor cortex. And the third one of them located at the medial part of the left parietal lobe. The two ones were extracted using stereotaxy*.

**Figure 7:**
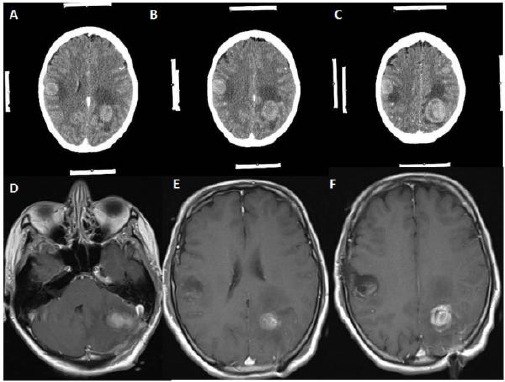
*A, B, C (Table 1: Case 5, Metastatic lung tumour): Contrasted axial computerized cranial tomography scans showing that hyperdense oval-shaped lesions with perilesional edematous area at the right frontal lobe and left frontal lobe. Both of these lesions were extracted using stereotaxy technique. D, E, F: Gadolinium-enhanced T1 weighted axial magnetic resonance image scans taken at the postoperative period showing no residual lesion, but there is some postoperative hyperintense changing due to gliosis at the operation regions. I restated that the lesion of the left side of the posterior fossa is not a data for this study, but this lesion also was extracted in gross total manner*.

**Figure 8:**
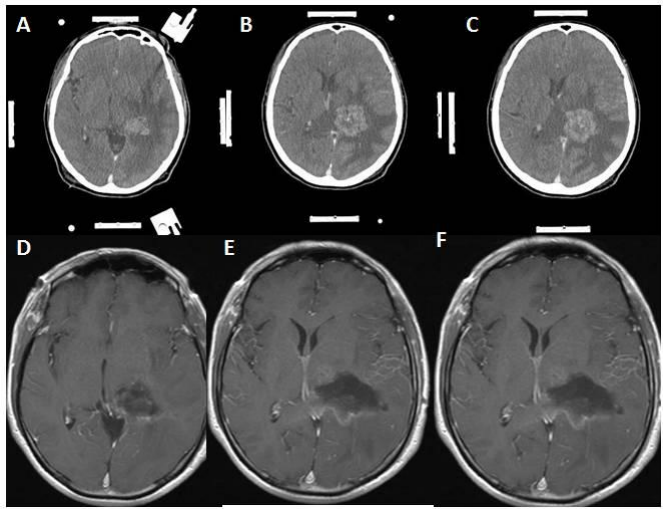
*A, B, C (Table 1: Case 8, Metastatic lung tumour): Contrast-enhanced cranial tomography showing a lesion located just next to the left thalamus with large perilesional edema. Moreover, this lesion showing contrasts enhancement and heterogeneous density. The white strips at the sides of the images are the marker of the frame based the stereotaxic system. D, E, F: Gadolinium-enhanced T1 weighted axial magnetic resonance image scans taken at the postoperative period showing no residual lesion, but there is some postoperative hyperintense changing due to gliosis at the operation regions*.

### Discussion

Using Advanced Technologies in neurosurgery provides less complication, less hospitalization period and more comfortable recovery period [9, 10]. These advanced technologies are CT compatible stereotaxic systems, frameless stereotaxic systems compatible with MRI. In addition to MRI, different modalities of the MRI are available, such as MRI tractography of the white matter (Diffusion-tensor imaging techniques for mapping of white matter tracts of the brain), functional MRI, intraoperative MRI.

Moreover, neuronavigation with advanced operative microscopes such as confocal laser endomicroscopy and microscope integrated indocyanine green video-angiography in cerebrovascular surgery [9-14]. Correspondingly, using new technologies in neurosurgical practice attracts the neurosurgeons also specifically patients who try to find best options for their treatment. However, it is not possible to be available every new kind of new technologies in every country and every hospital because of economic reasons. However, it is possible to achieve reasonable results combining cranial MRI scans and CT compatible stereotactical systems with microsurgical technique. There are some common points in neuronavigation system, MRI compatible stereotaxic system and CT compatible stereotaxic system.

Those are:

1 - Determination of the exact localization of the lesion is possible in three of the system. Nonetheless, MRI compatible stereotaxis is superior in taking biopsy in patients with lesion is not detectable in contrast-enhanced cranial CT than CT compatible stereotaxic systems. However, lesions are visible on cranial CT with or without contrast enhancement CT compatible stereotaxic systems provide almost equal positive biopsy results as to MRI compatible stereotaxic system.

Correspondingly, using CT compatible stereotaxic systems for localizing cranial tumours offer same advantages to the patients with lesions are visible with or without contrast-enhanced CT as to MRI compatible stereotaxis in localizing cranial tumours. These benefits: firstly, exact determination of the target point for removing the tumour in stereotactic craniotomy provides smaller craniotomy than conventional craniotomy. Secondly, we control our tumour target in three stages during stereotactical craniotomy. In the first phase, lesion targeted craniotomy provides to find the exact place of the tumour as being in all cases. Following craniotomy, the second stage of the operation starts. In this stage, stereotactical targeting shows us the center of the lesion, and we would be able to detect the entry point to reach the tumour. Knowing the exact entry point to the tumour provides us best trajectory, because we can change the target trajectories to reach the tumour. Moreover, this provides us reaching the tumour center without or minimal touching any cerebral cortex as in our case 1. Moreover, changing a trajectory through stereotactical system provides to protect eloquent regions of the cerebrum but it does not cause any changing of the central target of the tumour. This target constancy will provide us to protect eloquent cortical areas. After we calculate the target points from the cortex, it would be better transsulcal reaching the deeper site of the cerebrum to protect more cortical areas. Lastly, at this stage we would not need to make cortical incision find the tumour in the cerebrum if the tumour partly above the cortex as in our case 1. In the third stage that is specifically related to the tumours located deep site of the brain. Following transsulcal cortical incision, we would be able to reach deeper part of the tumour through stereotactic system as in our case 4 and case 6. Using stereotaxy into deeper parts of the brain would also help us to distinguish between gliosis and tumour because we pursue the center of the tumour.

2 - Exact localization would also reduce the retraction of the cerebrum by cerebral retractor is a surgical instrument by which a surgeon can either actively separate the edges of cerebral cortical incision and can hold back underlying cerebral tissue. In conventional craniotomy, more cortical incision and more retraction would be necessary to find and to remove the tumor. Therefore, the increased incision size and retraction would cause tearing of the cortical and subcortical structures. Along with neuronal and axonal damage related with the retraction and increased incision size, higher pressure to seperate the cerebral tissue would block venous drainage. All of these events would initiate arterial obstruction and hemorrhagic necrosis and hemorrhage of the parenchyma of the cerebrum. In addition to these damages, inadvertent tearing of the venous and arterial vasculature would occur. For example, in case 1, we were able to remove the tumor just behind the motor cortex among the venous structures without causing any damage to the cerebrum (Fig. 1, 2). Case 1 was also an excellent example of removing the tumour without making any cortical incision and retraction to the cerebrum.

3 - Exact localization would also provide smaller craniotomy regarding conventional craniotomy as in case 5 (Fig. 6, 7). Nonetheless, making small craniotomy for the lesion with large edema region and located around the motor cortex and parasagittal region would be dangerous because of incarceration of the cerebrum following craniotomy and opening of the duramater. Because significant edema causes increasing of the intracranial pressure, and increasing of the intracranial pressure leads to cerebral ischemia. Consequently, small craniotomy and small flap of the duramater would cause to be pushed off the cerebral tissue from the inside to the outside because of increased intracranial pressure, and, within minutes, the edges of the duramater would act like a muff. Moreover, duramater edges cause more positive pressure within at the protruded part of the cerebrum (Fig. 1, Fig. 8). To prevent incarceration of the brain, at least, in some cases, tumours have a large edematous region, and located eloquent areas should be evaluated for making more extensive craniotomy and larger duramater flap, because eloquent areas of the cerebrum are rich in arteriovenous vasculature and correspondingly drainage veins (Figures 1 & 8).

Different authors advocated that using of stereotaxic surgery [5, 6, 15-17]. However, the various kind of stereotaxis and neuronavigation systems would cause confusion among the neurosurgeons toward this technique, for example, using tubular retractor system with stereotaxy, volumetric stereotaxy, frameless stereotactic resection and neuronavigation with tractography, intraoperative MRI [4, 9-13, 16, 17]. All of this techniques have advantages to localizing the lesion within the brain. However, it is not possible to be available all of them in one institute.

Greenfield and his colleagues have used frameless tubular retraction system. Moreover, they asserted that tubular retraction system with frameless stereotaxy for removing deep- seated brain lesion have provided few advantages. These are protection vascular structure, controlled application of the retraction with tubular retractor system¬, which provides equal pressure distribution around the tubular system and lesser damage to the cerebral cortex [17]. Moreover, Russel and Kelly, New York University School of Medicine have used Computer Assisted Volumetric Stereotaxy for the lesions located posterior part of hippocampus and parahippocampal gyrus [16]. Regarding their article, they think that this approach provides more than one advantages, including prevention of the lateral temporal and occipital lobe tracts injuries, avoidance of unnecessary brain resection and retraction[16]. Also, various authors using different stereotactic methods to find and to excise the lesion at brain [5-7, 18]. Surgical results of this stereotactic methods showing much better clinical outcomes and more amount of lesion resection than conventional craniotomy [5-7, 16-18]. The problem is which one is more effective than the others. From my perspective, as a neurosurgeon performing stereotaxic biopsy and stereotaxic craniotomy with arc-based CT compatible stereotactic frame system (Fischer ZD, Germany), I explain a few reasonable reasons to support this system. Firstly, this system and its application is simple. Moreover, it is effective to locate the lesion and also to reach the deepest part of the cerebrum for removing lesion, including glioma, meningioma, cavernoma, abscess regarding in our presented cases. For example, in this article we presented eight cases, and we achieved gross total resection confirmed with preoperative and postoperative CT and MRI. As well as we introduced the CT and MRI figures of particular instances in this article, we did not encounter any complication, such as hemorrhage or inadvertent injury to the vascular or neural structure in these cases. Secondly, application of the headholder and transfer of the patient to CT unit for taking of the cranial CT. Then, we move the patient to the operation theatre, and calculation of the coordinates for lesion targetting and fixing the patient having headholder to the operation room takes 30 minutes in our institute. Thirdly, we compare our CT results with patients’ contrasted MRI. We decided about craniotomy and cortical incision and to advance deeper part of the brain with the guidance of the CT stereotaxic system. Fourthly, we make shorter cortical incision and lesser brain retraction to reach the lesion located in the brain. Also, we can select the intersulcal region, the safest place to incise the cortex of the cerebrum, to reach the lesions in case 5 and case 6 (fig. 6-8). Moreover, we attained the tumour without making incision to the cortex in case 1 (fig 1, 2, 3). Fifthly, All of the patients were discharged within seven days, and they were in better neurologic condition. The last but not least, in deep- seated lesion, we have a chance to make discrimination between gliosis and lesion. All of these reasons may be enough to show the effectivity of the CT compatible arc based stereotaxic system. So we offer to use this system in patients with cranial tumour located eloquent areas or deep parts of the brain to protect inadvertent injury to neural structures and to shorten operation time and recovery period of the patient.

In conclusion, neurosurgeons provide gross total resection in critical brain tumors by using an arc based CT compatible stereotaxic system (Fischer ZD, Germany) and microsurgery techniques. In addition, they prevent further deficits during extirpation of the tumor and would provide improvement in developed neurologic deficits in critical brain tumors located either at the eloquent areas or at deep parts of the brain. Lastly, with this technique, neurosurgeons provide shorter recovery period and early discharge from the hospital to their patients.
